# *In situ* heavy ion irradiation studies of nanopore shrinkage and enhanced radiation tolerance of nanoporous Au

**DOI:** 10.1038/srep39484

**Published:** 2017-01-03

**Authors:** Jin Li, C. Fan, J. Ding, S. Xue, Y. Chen, Q. Li, H. Wang, X. Zhang

**Affiliations:** 1 Department of Materials Science and Engineering, Texas A&M University, College Station, TX 77843-3123, USA; 2 School of Materials Engineering, Purdue University, West Lafayette, IN 47907, USA; 3 Department of Mechanical Engineering, Texas A&M University, College Station, TX 77843-3123, USA; 4 MPA-CINT, Los Alamos National Laboratory, Los Alamos, NM 87545, USA; 5 Department of Electrical and Computer Engineering, Texas A&M University, College Station, TX 77843-3128, USA

## Abstract

High energy particle radiations induce severe microstructural damage in metallic materials. Nanoporous materials with a giant surface-to-volume ratio may alleviate radiation damage in irradiated metallic materials as free surface are defect sinks. Here we show, by using *in situ* Kr ion irradiation in a transmission electron microscope at room temperature, that nanoporous Au indeed has significantly improved radiation tolerance comparing with coarse-grained, fully dense Au. *In situ* studies show that nanopores can absorb and eliminate a large number of radiation-induced defect clusters. Meanwhile, nanopores shrink (self-heal) during radiation, and their shrinkage rate is pore size dependent. Furthermore, the *in situ* studies show dose-rate-dependent diffusivity of defect clusters. This study sheds light on the design of radiation-tolerant nanoporous metallic materials for advanced nuclear reactor applications.

Energetic particle (such as ions, neutrons and protons) radiations induce a large population of vacancies and self-interstitial atoms (SIAs) in metallic materials, and these point defects can combine into clusters, in the form of dislocation loops and networks, voids, bubbles and stacking fault tetrahedra (SFTs)^1^,^2^,^3^,^4^. As a result of severe radiation, the microstructure and mechanical properties of the materials can be significantly degraded as evidenced by void swelling, radiation hardening, embrittlement and significant loss of ductility^5^,^6^,^7^,^8^,^9^. The search for radiation tolerant materials has been a subject of intense research for decades. Numerous approaches have been adopted to design radiation-resistant materials. The central premise is that certain types of defect sinks may promote the absorption and recombination of interstitials and vacancies and thus enhance the radiation tolerance of materials. Consequently, various types of defect sinks have been investigated, including grain boundary (GB), twin boundary (TB), phase boundary, etc^10^,^11^,^12^,^13^,^14^,^15^,^16^,^17^,^18^. Nanocrystalline materials have abundant GBs and may have extraordinary radiation resistance because GBs act as defect sinks that can absorb irradiation induced defects, and in some cases may serve as sources to emit interstitials and annihilate vacancies^19^,^20^,^21^,^22^,^23^. TBs are low energy boundaries and are generally considered to be weak defect sinks. However, recent studies reported that TBs in nanotwinned (nt) metals can capture and rapidly transport defect clusters, remove irradiation-induced defects (including SFTs) effectively, form a TB affected zone within which the accumulative defect cluster density is lower than that in crystal interior, and thus nt metals may have remarkable radiation resistance^20^,^24^,^25^. Layer interface is another effective defect sink and its influence on radiation tolerance of materials has been extensively studied^14^,^15^,^17^,^26^. For instance, significant layer thickness-dependent reduction of both helium bubble density and alleviation of radiation hardening were reported in some of the immiscible systems, such as Cu/Nb^27^ and Cu/V^28^. MD simulations of Cu/Nb also indicate that the Cu-Nb interface is a highly efficient sink for the annihilation of irradiation-induced defects^29^.

Free surface is typically considered as an unsaturable defect sink, and nanoporous (np) materials with a giant surface-to-volume ratio may have enhanced radiation tolerance compared to the fully dense coarse-grained (cg) counterparts^30^,^31^,^32^. There are extensive studies on mechanical properties of np metals^33^,^34^,^35^,^36^. However, there are limited cases on irradiation responses of np materials^12^,^31^. *Ex situ* study on np Au under 400 keV Ne^++^ ion irradiation shows that defect accumulation depends on dose rate^37^. More specifically, SFTs form at high dose rate, while few SFTs are generated at low dose rate. Their MD simulations indicate that low dose rate leaves ample time for SIAs and vacancies to diffuse to the surface or recombine, whereas at higher dose rate, the time interval between cascades is shorter than the time needed for migration of defects to the surface. Consequently, vacancies have enough time to aggregate and form SFTs. Previous *in situ* study on np Ag shows the removal of various types of defect clusters, including SFTs, small dislocation loops and large dislocation segments, by the free surface in np Ag under 1 MeV Kr^++^ ion irradiation^32^. Furthermore, the *in situ* study shows that both the global and instantaneous diffusivities in np Ag are lower than those in cg Ag.

Despite these findings, the number of *in situ* studies on irradiation response of np metals remains scarce. In addition, defect migration kinetics, which is crucial for the modeling of defect evolution in irradiated metals, remains largely unknown^38^,^39^. The influence of dose rate on defect diffusivities is still unclear^2^,^31^,^40^.

Here we present *in situ* Kr ion irradiation studies on cg and np Au. The migration of defect clusters and their elimination by free surfaces are captured by *in situ* video. Our studies show^32^ that np Au has significantly better radiation tolerance than coarse-grained Au in terms of reduced size and density of radiation-induced defect clusters. Furthermore, we report on the dose-rate-dependent defect global and instantaneous diffusivities in np Au, which has not been studied in np Ag previously. In addition, nanovoids shrink due to the absorption of defect clusters by nanovoids during irradiation in np Au, and their shrinkage rate depends on pore size. The outstanding irradiation tolerance of np Au has important implications for the design of advanced np materials under extreme radiation environment.

## Results

**Figure 1:**
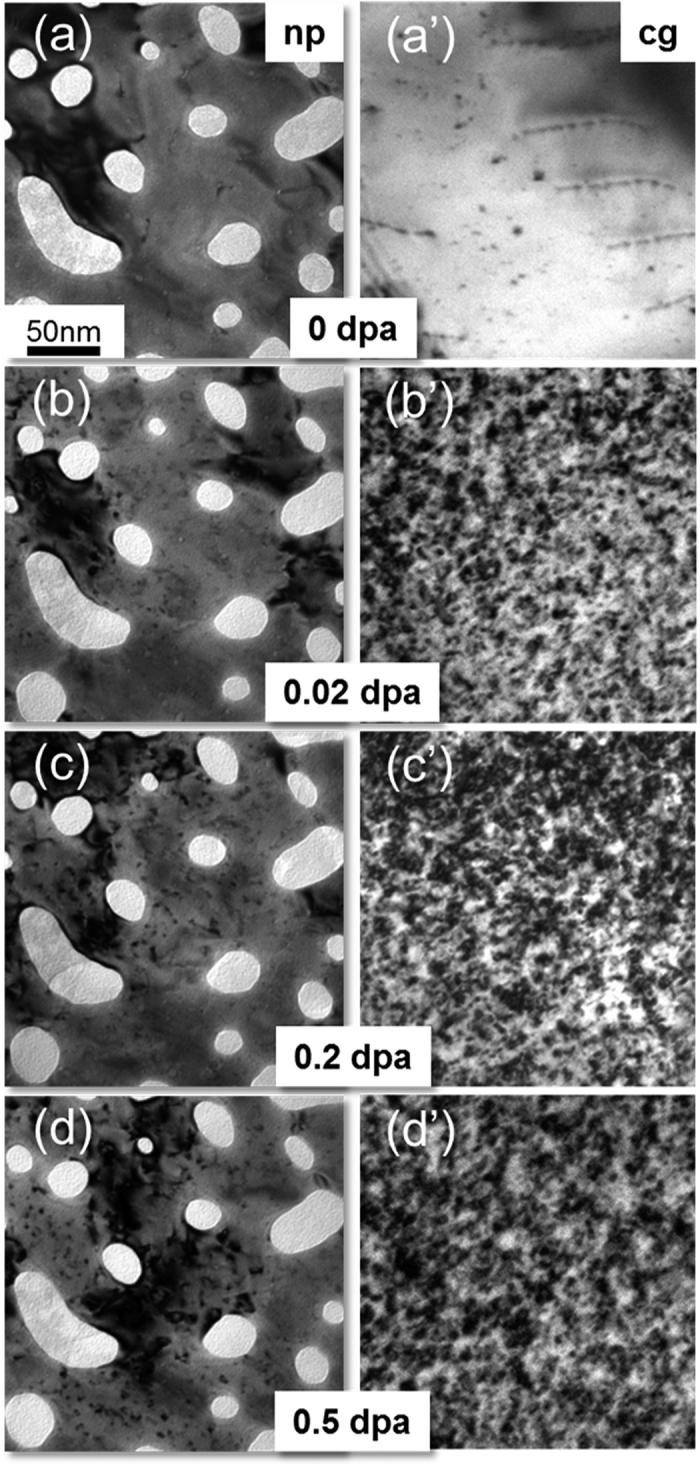
Transmission electron microscopy (TEM) snap shots obtained from *in situ* radiation video revealed drastically different irradiation response between nanoporous (np) and coarse grained (cg) Au subjected to *in situ* Kr ion irradiation at room temperature. (**a**-**a**’) Before irradiation, both np Au and cg Au appeared relatively clean with few preexisting defects. (**b**–**d**) TEM snap shots show gradual and moderate increase of defect density in irradiated np Au, up to 0.5 dpa. (**b**’–**d**) In contrast, cg Au has accumulated much more defects rapidly by 0.5 dpa.

**Figure 2:**
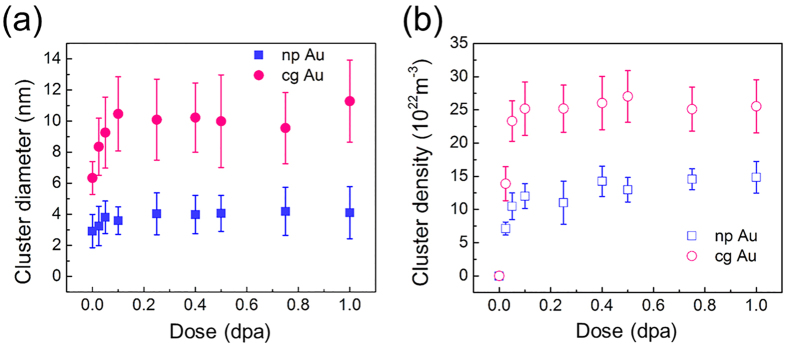
Statistics of defect cluster diameter and density in np Au and cg Au. (**a**) The average defect diameter is ~10 and 4 nm for cg and np Au, respectively. (**b**) The defect density in both cg and np Au reached saturation at similar dose level, ~0.1 dpa.

As-prepared cg Au and np Au were both transparent to the electron beam. The diameter of nanovoids varies from ~10 to 100 nm, and the selected area diffraction (SAD) pattern suggests the film was highly textured (see Suppl. Fig. S1a). Figure 1 compares the microstructural evolution in cg and np Au under 1 MeV Kr^++^ ion irradiation at room temperature. Before irradiation, both np Au and cg Au had little preexisting defects (Fig. 1a–a’). Radiation leads to a gradual and moderate increase of defect density in np Au, up to 0.5 dpa (Fig. 1b–d). In contrast, cg Au has accumulated much more defects rapidly by 0.5 dpa (Fig. 1b’–d’). Bright field and weak beam dark field (WBDF) TEM images show that a majority of defect clusters are dislocation loops and SFTs (Supp. Fig. S1b–d). TEM snapshots from *in situ* videos compare cg and np Au irradiated to several doses. From 0 to 0.02 dpa (Fig. 1b and 1b’), few defect clusters formed in np Au, whereas defect density in cg Au increased rapidly (Supp. Video 1: 0–0.02 dpa). By 0.2 dpa, both the diameter and density of defect clusters in cg Au increased significantly (Fig. 1c’), while only a few defect clusters were generated in np Au (Fig. 1c and Supp. Video 2: 0.15–0.2 dpa). By 0.5 dpa (Fig. 1d and d’), the average diameter of defect clusters in np Au appeared much smaller than that in cg Au. Figure 2 shows the statistics of the diameter and density of defect clusters in np Au and cg Au. The average defect size is ~10 and 4 nm for cg and np Au, respectively (Fig. 2a). The defect density in both cg and np Au reached saturation at similar dose, ~0.1 dpa (Fig. 2b).

**Figure 3:**
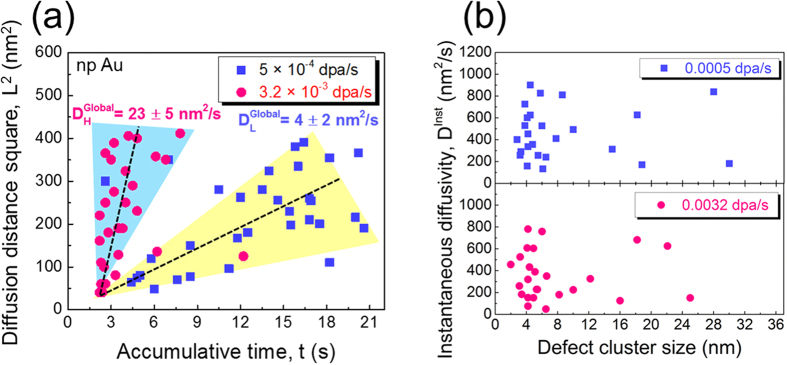
The global and instantaneous diffusivities of defects under different dose rate in np Au. (**a**) The global diffusivity of defects in np Au is significantly reduced (by six times) when the dose rate decreased from 3.2 × 10^−3^ to 5 × 10^−4^ dpa/s. (**b**) The instantaneous diffusivity of defect clusters in irradiated np Au varies from 200 to 800 nm^2^/s, and the average value of instantaneous diffusivity shows little dependence on dose rate and cluster diameter.

The migration of a large number of defect clusters in np Au has also been examined to estimate their global and instantaneous diffusivities. The global diffusivity, D^Global^, of a defect cluster is determined by using the square of overall defect migration distance (L^2^) over the accumulative defect lifetime, which includes the migration time and dwelling time. Most defects migrate in a ‘stick-slip’ manner, that is a defect cluster migrates instantaneously within a fraction of a second, and then stays for a while (dwelling time) before its next movement. Thus D^Global^ is correlated qualitatively to the average migration speed of a defect cluster over its entire lifetime. Meanwhile, we have also determined the instantaneous diffusivity, D^Inst.^, of a defect cluster by only considering the diffusion distance over its migration time (excluding dwelling time). Thus D^Inst.^ appears much greater (by an order of magnitude) than D^Global^. The detailed methods to determine diffusivity have been shown previously^25^. Figure 3a shows D^Global^ of defect clusters in np Au at a higher dose rate of 3.2 × 10^−3^ is ~23 ± 5 nm^2^/s (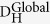), significantly greater than ~4 ± 2 nm^2^/s at a lower dose rate of 5 × 10^−4^ dpa/s (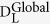). Meanwhile, Fig. 3b shows the D^Inst.^ of defect clusters of irradiated np Au varies from 200 to 800 nm^2^/s, and due to a large scattering, the average value of D^Inst.^ shows little dependence on dose rate.

**Figure 4:**
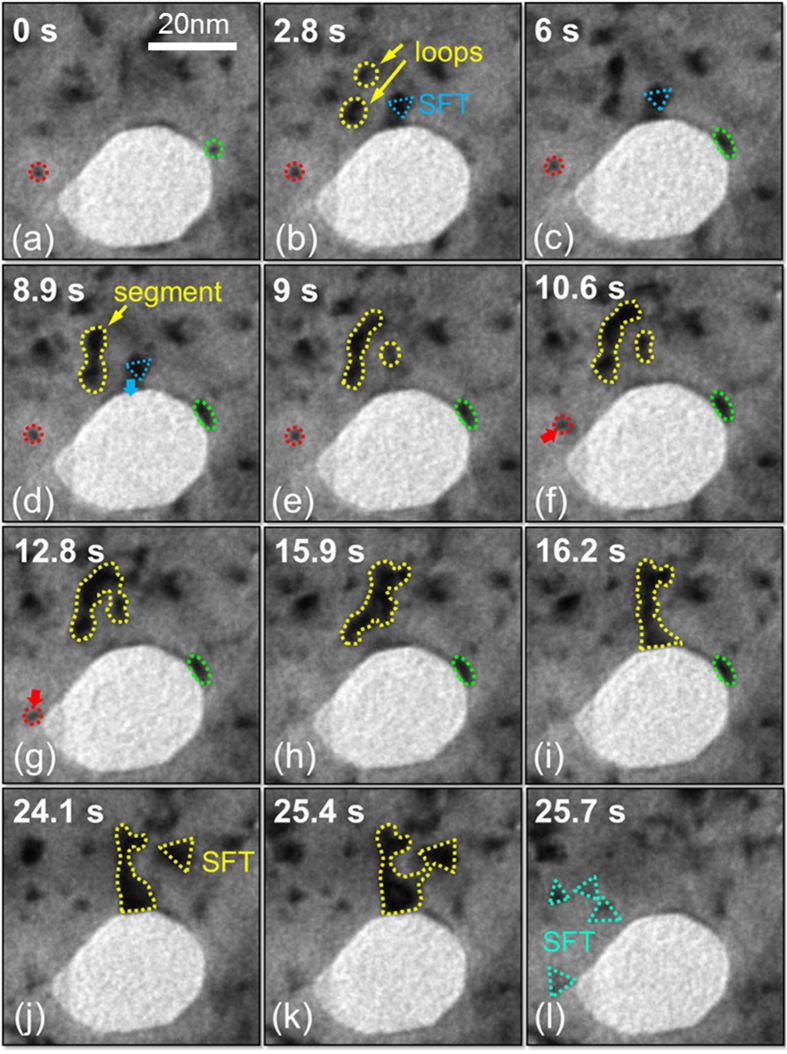
*In situ* video snapshots showing several representative defect capture events by an individual nanovoid over 0.02–0.04 dpa (~26 s). (**a**) Two small isolated loops were observed, one of them (outlined by red dots) was several nm away from the nanovoid, and the other one was adjacent to the void (green marker). (**b**) After 2.8 s, two more loops (indicated by the yellow markers) and a SFT (blue marker) emerged near the void. (**c**–**e**) The individual loops combined into a short dislocation segment. Meanwhile, the SFT interacted with the void surface and was destructed. (**f**–**h**) A small dislocation loop (marked by the red dotted line) migrated towards the void and was captured by the void at 15.9 s. (**i**–**l**) The dislocation segment and a SFT interacted with each other and were eventually eliminated by the adjacent void.

Typical examples of the evolution and interaction of defect clusters with nanovoids are demonstrated by *in situ* video snapshots over 0.02–0.04 dpa in Fig. 4 (Supp. Video 3). Two small isolated loops were identified at 0 s, one of them (outlined by red dots) was several nm away from the nanovoid, and the other one was adjacent to the void (marked in green) (Fig. 4a). After 2.8 s, two other loops (indicated by the yellow dotted lines) and an SFT (blue marker) emerged near the void (Fig. 4b). Figure. 4c–e show the individual loops combined into a dislocation segment. Meanwhile, the SFT interacted with the void surface and was destructed. In Fig. 4f–h, the same video also shows a small dislocation loop (marked by the red dotted line) migrated towards the void and was captured by 15.9 s. The dislocation segment combined more defects and then interacted with an adjacent SFT, leading to their mutual destruction (Fig. 4i–l). Later a cluster of small SFTs emerged at 25.7 s during irradiation (Fig. 4l).

**Figure 5:**
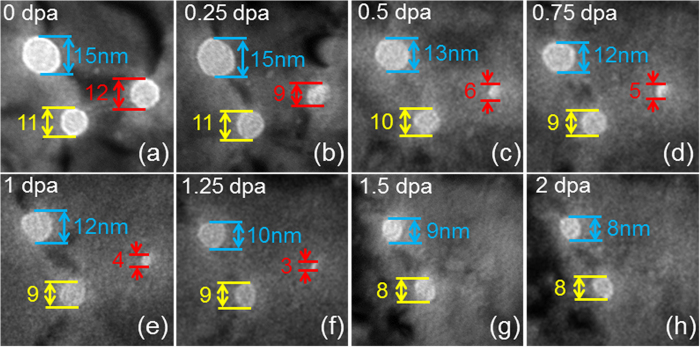
*In situ* video snapshots showing the shrinkage of nanopores during irradiation (0–2 dpa). (**a**) Three pores with diameter of 15, 12 and 11 nm were observed before irradiation (0 dpa). (**b**–**e**) During irradiation, a large number of defects migrated to the pores (defect absorption), and the dimension of pores continuously decreased. (**f**) By 1.25 dpa, the diameter of the void (marked by red arrows) decreased substantially from 12 to merely 3 nm, and finally disappeared by 1.5 dpa (**g**). (**h**) The diameter of the other two nanopores changed from 15 to 8 nm and from 11 to 8 nm, separately.

**Figure 6:**
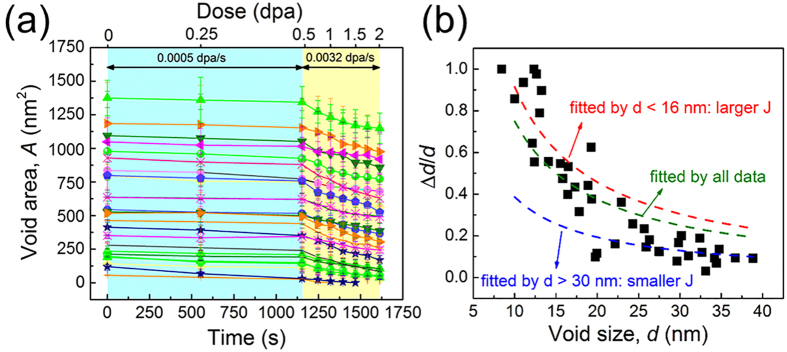
Statistic data revealing the shrinkage of nanopores during irradiation (0–2 dpa). (**a**) Statistic data showing the evolution of pore diameter vs radiation dose. Nanopores shrank much faster at higher dose rate, 0.0032 dpa/s, compared to lower dose rate, 0.0005 dpa/s. (**b**) The normalized diameter reduction, Δ*d*/*d*, as a function of pore size. The olive dashed line is the result of fitting for all data, and the red dashed line and blue dashed line are fitting results by choosing the data in the range of d < 16 nm and d > 30 nm, respectively. A significant deviation between three fitting results indicates that *J* is greater (smaller) for smaller (larger) voids.

Another phenomenon is the universal shrinkage of numerous nanopores during *in situ* radiation of np Au and an example is shown in Fig. 5. Three pores with diameters of 15, 12 and 11 nm were tracked during radiation over 2 dpa (~1600 s). During irradiation, a significant number of defect clusters migrated to these pores (defect absorption), and the dimension of pores decreased continuously (Fig. 5a–e). For instance, by 1.25 dpa (Fig. 5f), the diameter of the pore (marked by red arrows) decreased substantially from 12 to merely 3 nm, and eventually disappeared by 1.5 dpa (Fig. 5g). The diameter of the other two nanopores changed from 15 to 8 nm, and 11 to 8 nm, respectively over 2 dpa. Statistical data in Fig. 6a show that nanopores shrink much faster at higher dose rate, 3.2 × 10^ − 3^ dpa/s, compared to lower dose rate, 5 × 10^− 4^ dpa/s. Furthermore, the normalized shrinkage of pores, Δ*d*/*d*, decreases rapidly with increasing pore size (Fig. 6b), indicating smaller pores shrink faster during irradiation.

## Discussion

Free surfaces are perfect sinks for defects, so it is natural to speculate that np Au with high surface-to-volume ratio would be more radiation tolerant compared to its cg counterpart. Indeed, our *in situ* studies show np Au has excellent radiation resistance as evidenced by its substantially lower defect dimension and density than those in cg Au. During irradiation, although the defect density in both cg and np Au reached saturation at similar dose level, the continuous migration of point defects and their clusters towards nanovoids leads to significant reduction of defect concentration, manifested by an overall decrease in defect size and density. Although it appears that there is only a moderate reduction of defect cluster density (a factor of 2) and cluster size (a factor of ~2.5) for np Au compared to cg Au, the point defect concentration difference can be substantial. Assuming all defect clusters are spherical, the point defect concentration amounts to (2.5)^3^ × 2 ≈ 30. Furthermore, many of the defect clusters in cg Au are much greater than the average defect diameter, whereas the defect clusters in np Au has a much narrower size distribution. *In situ* studies show the capturing of numerous types of defect clusters (including SFTs, individual dislocation loops, and dislocation segments) by nanovoids. The removal of various types of defect clusters by free surface has been reported previously during *in situ* radiation study of np Ag^32^.

Another significant observation revealed by *in situ* radiation study is the shrinkage of nanovoids in np Au at room temperature. During radiation, most of the vacancies are bound in the form of sessile defect clusters, and thus there may be insufficient vacancies to support void growth. Meanwhile, there is a continuously biased flock of interstitials and their clusters to nanovoids during irradiation. As void shrinkage in the current study is caused by the absorption of irradiation-induced defects, these deliberately introduced nanovoids are effective defect sinks, providing np Au much better radiation tolerance than their cg counterparts. Furthermore, it is natural to speculate that the void shrinkage rate could be determined by the defect capture (absorption) rate or the frequency of interactions between nanovoids and defects. Figure 6a confirms this hypothesis by tracking the evolution of diameters for a number of nanovoids. When the dose rate is increased by ~ six times, the void area, *A*, decreased much faster. Void shrinkage rate (indicated by the slope of the plots) in the high dose rate area (shaded by yellow color) is ~ six-times larger than the slope of the data in low dose rate area (highlighted in blue color), indicating the void shrinkage rate scales with dose rate.

Besides the direct observation of defect absorption events, another benefit of *in situ* study is that it allows us to estimate global and instantaneous diffusivities of defect clusters by carefully analyzing the videos and tracking the migration of defect clusters continuously during irradiation. More details on the methods to estimate diffusivity can be found in previous studies^24^,^25^,^32^. The global diffusivity for defect clusters in np Au is calculated to be 4 ± 2 nm^2^/s when the dose rate is 5 × 10^−4^ dpa/s, and 23 ± 5 nm^2^/s at a higher dose rate of 3.2 × 10^−3^ dpa/s. Clearly, the global diffusivity is significantly reduced (by six times) when the dose rate decreases. However, the instantaneous diffusivity in np Au varies from 200 to 800 nm^2^/s and shows little dependence on dose rate. Such intriguing phenomena clearly warrant further discussions.

Both global and instantaneous diffusivities are determined by two factors: diffusion distance and a defect’s lifetime. In this study, the diffusivities of defect clusters were estimated when defect density reached saturation. The saturated defect cluster density in np Au is ~15 × 10^22^ m^−3^ as shown in Fig. 2b, and therefore, the distance between two defect clusters can be simply estimated to be 15 ~21 nm (these values considered (1) average defect diameter, 4 nm, and (2) the length between two defect clusters can be either edge length or diagonal length in a square). Although the dose rate has been changed, the defect density has reached a plateau (independent of dose rate from ~0.1–2.0 dpa). It follows that the average diffusion distance (limited by defect separation distance) for both high and low dose rate is nearly the same, ~18 nm (see Fig. 3a). Thus the global diffusivity of defects may be largely determined by defects’ lifetime.

A defect’s lifetime includes two components, defect migration time (hopping time) and dwell time (rest time). To estimate the global diffusivity, both migration and dwell time were taken into account. As a defect migrate very rapidly, the lifetime of a defect is dominated by its dwell time. Supplementary Video 4 shows the drastic difference in defect migration activity in np Au irradiated at low and high dose rate. Compared to low dose rate, both defect generation rate and recombination rate increase prominently at higher dose rate. As a consequence, a defect cluster cannot dwell for a long time. Instead, it will frequently migrate toward the free surface, or combine with other clusters. Thus the defect’s dwell time significantly decreases at higher dose rate. Since diffusion lengths are similar in both cases (low vs. high dose rate), the global diffusivity at high dose rate is larger than that at low dose rate.

In contrast to global diffusivity, only a defect’s migration time is considered to determine the instantaneous diffusivity. Nearly all defects migrated within a single frame, and therefore, a defect’s migration time is assumed to be 66 ms for a single migration event (limited by the camera’s capture speed of 15 frame/s). Notice that such an assumption may overestimate a defect’s migration time. There is a large scattering of instantaneous diffusivity. Consequently, the instantaneous diffusivity appears to have an insignificant dependence on dose rate and defect dimension.

We now examine the shrinkage of nanovoids in np Au. Void swelling is frequently observed in many neutron and heavy ion irradiated metals at elevated temperatures^41^,^42^,^43^,^44^,^45^, while void shrinkage is commonly observed at room temperature or at the temperature much below the material’s melting point^46^,^47^,^48^,^49^. It is known that vacancies will be bundled in thermally stable defect clusters, such as vacancy loops and SFTs, during energetic displacement cascade under heavy ion irradiation, and interstitials are mobile near room temperature^50^,^51^,^52^. Therefore, void shrinkage may be anticipated in the current study. However, a question remains to be addressed is: Does defect absorption efficiency dependents on void size in np materials? Figure 6b may provide some clues to this question.

Assuming the incoming flux of defects into a single void is a constant *J* (unit: volume/area·second), and these defects will fill in the voids (leading to the reduction of void diameter), then *J* can be simply estimated by (assuming that most nanovoids are through thickness nanopores with cylindrical shape):


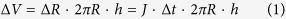


where *V* is the volume of voids, *R* (=*d*/2) is the radius of voids, *h* is the film thickness. Δ*R* is the reduction of void radius over a period of Δt. It is readily seen thatΔ*R* = *J*Δ*t*, i.e., Δ*d = *2*J*Δ*t*. Consequently the relation between normalized void shrinkage, Δ*d*/*d*, an*d* void size *d* can be expressed as:


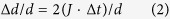


Thus, the normalized void shrinkage is inversely proportional to the initial defect diameter. This is very close to what has been captured experimentally (Fig. 6b). Furthermore, the attempt to use a constant J to fit all data point (Olive dash line) seems to underestimate (overestimate) Δ*d*/*d* for smaller (larger) voids. Such systematic deviation suggests that *J* is greater for smaller voids and less for larger voids. In other words, defects may interact more frequently with smaller voids than the larger ones during irradiation, and therefore, defect absorption efficiency is higher for smaller voids than larger voids. In a recent study on nanovoid-nanotwinned Cu (with an average void diameter of ~10 nm), significant loop-void interactions were observed. Analytical calculations show the existence of significant tensile stress surrounding voids^53^. When a loop moves closer to a void, its migration rate increases drastically due to the substantial reduction of formation and migration energies of the loop under tensile stress. Compared to larger voids, smaller voids generate higher stress field near void surfaces and therefore, smaller voids capture defects more rapidly during irradiation.

It may appear that the irradiation resistance of np metals will degrade over the long term if most of the nanovoids are completely filled with radiation induced defect clusters. Such concern can be eased for the following reasons. First, bulk (fully dense) Au is extremely vulnerable to radiation damage as shown by severe radiation damage at merely 0.02 dpa. It is clear that nanovoids significantly delay damage accumulation in Au by more than an order of magnitude, rendering np Au resistance to heavy ion irradiations to a much greater dose (1 dpa). Furthermore, the foregoing studies show that the concentration of point defects (such as SIAs) could also be an order of magnitude lower in np Au than that in cg Au. Such a concept derived from the current study indicates that the safe operation period (lifetime) of nuclear reactor steels, if engineered with nanoporous structures, could also be significantly extended. Second, Au is a model system that is known to be vulnerable to radiation damage than many other alloys, such as austenitic stainless steels. One can envision that for a practical reactor steel, the incorporation of nanovoids/nanopores could achieve even greater radiation tolerance than what has been demonstrated in the current model system. Third, although nanovoids may be eliminated during radiation, modeling tool (such as phase field models), may reliably predict the correlation between the service lifetime and desirable combination of different diameter and density nanovoids for design purposes. As Fig. 6(b) suggested, smaller voids shrink faster than the larger ones. Therefore, by deliberately introducing nanovoids with different diameters and density, the radiation stability of reactor steels may be significantly prolonged. Of course, further improvements in nanopore stability might also be beneficial before this concept transfers into a practical option for future reactor steels.

## Conclusion

*In situ* heavy ion irradiation studies were performed on nanoporous Au at room temperature. Dose-rate-dependent defect migration diffusivities were examined, and the global diffusivity is significantly reduced when the dose rate decreases, while the instantaneous diffusivity shows little dependence on dose rate. Nanovoids are effective defect sinks where various types of defect clusters can be absorbed during irradiation. The absorption of defect clusters leads to the shrinkage of nanovoids, and the shrinkage rate, or defect absorption efficiency, is size-dependent. Comparing to larger voids, smaller voids exhibit higher defect absorption efficiency. This study provides significant insight into the design of radiation-tolerant nanoporous metallic materials, such as nanoporous reactor steels for advanced nuclear reactors.

## Method

The Ag_65_Au_35_ (atomic ratio) leaves (procured from New York Central Art Co.) with dimensions 20 mm × 20 mm × 120 nm were sandwiched by two 304 stainless steel plates and then cold rolled up to ~20% strain so as to reduce the foil thickness and achieve electron beam transparent specimens. The rolled Ag_65_Au_35_ leaves were then chemically de-alloyed in a 70% HNO_3_ solution for 4 h at room temperature. The etched leaves were repeatedly rinsed in deionized water to remove residual acid and eventually lifted off by Cu grids (400 mesh) for *in situ* radiation studies. All specimens were investigated using an FEI Tecnai G2 F20 ST microscope before and after irradiation. *In situ* irradiation experiment was performed at room temperature at the IVEM-TANDEM facility at Argonne National Laboratory. An 1 MeV Kr^++^ ion beam was used for radiation experiments to a maximum fluence of 2 × 10^14^ ions cm^−2^ (∼1 dpa). The dose rate applied during *in situ* radiation experiments varied from 3.2 × 10^−3^ to 5 × 10^−4^ dpa/s. SRIM (Kinchin-Pease method) simulation was used to estimate the displacement damage profile (in the unit of displacements-per-atom (DPA)) and Kr ion distribution. Most Kr ions (99.99%) penetrated directly through the specimen and the residual Kr ion concentration in the TEM thin foil is ~0.01 at.%. The temperature rise of specimens during *in situ* Kr ion irradiation measured by thermocouple is less than 10 °C.

## Additional Information

**How to cite this article**: Li, J. *et al. In situ* heavy ion irradiation studies of nanopore shrinkage and enhanced radiation tolerance of nanoporous Au. *Sci. Rep.* **7**, 39484; doi: 10.1038/srep39484 (2017).

**Publisher's note:** Springer Nature remains neutral with regard to jurisdictional claims in published maps and institutional affiliations.

## Supplementary Material

Supplementary Video 1

Supplementary Video 2

Supplementary Video 3

Supplementary Video 4

Supplementary Figure and Video Legends
