# Supplementary material for: In situ heavy ion irradiation studies of nanopore shrinkage and enhanced radiation tolerance of nanoporous Au
Source: Sci Rep. 2017 Jan 3;7:39484. doi: 10.1038/srep39484 (PMC5206729; doi:10.1038/srep39484)
Supplement: Supplementary Figure and Video Legends [file srep39484-s5.pdf]

In situ heavy ion irradiation studies of nanopore shrinkage and enhanced radiation tolerance of nanoporous Au

Jin Li[a], C. Fan[a], J. Ding[a], S. Xue[b], Y. Chen[c], Q. Li[a], H. Wang[d] and X. Zhang[a, e]

[a] *Department of Materials Science and Engineering, Texas A&M University, College Station, TX 77843-3123, USA*

[b] *Department of Mechanical Engineering, Texas A&M University, College Station, TX 77843-3123, USA*

[c] *MPA-CINT, Los Alamos National Laboratory, Los Alamos, NM 87545, USA*

[d] *Department of Electrical and Computer Engineering, Texas A&M University, College Station, TX 77843-3128, USA*

[e] *School of Materials Engineering, Purdue University, West Lafayette, IN 47907, USA*

*Corresponding author: X. Zhang, xzhang98@purdue.edu

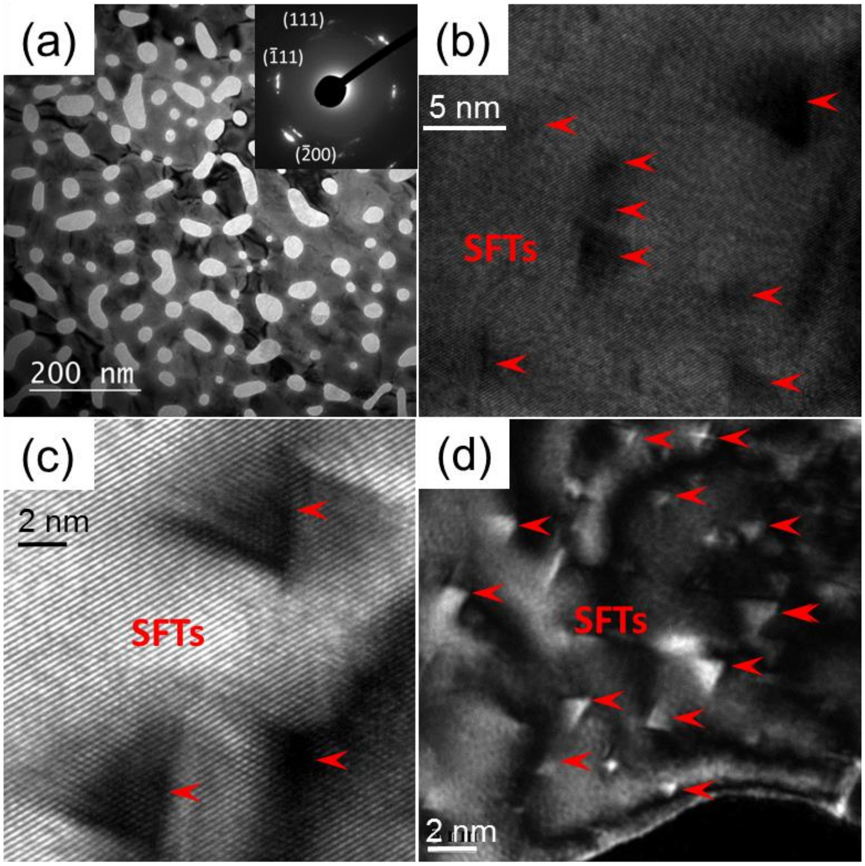

Fig S1. (a) Overview of porous structure of np Au. The inserted SAD pattern shows np Au exhibits (110) texture. (b) A bright-field and (c) a high-resolution TEM images show the existence of SFTs after irradiation. SFTs are the major type of defect clusters introduced during irradiation, and it's confirmed by a dark-field TEM image in (d). Most of the defect clusters in the image are SFTs.

Video Legends

Supplementary Video 1. Comparison between cg and np Au irradiated during 0 – 0.02 dpa. Few defect clusters formed in np Au, whereas defect density in cg Au increased rapidly.

Supplementary Video 2. Comparison between cg and np Au irradiated during 0.15 – 0.2 dpa dpa. By 0.2 dpa, both the diameter and density of defect clusters in cg Au increased significantly, while only a few defect clusters were generated in np Au.

Supplementary Video 3. The evolution and interaction of defect clusters with nanovoids over 0.02 – 0.04 dpa.

Supplementary Video 4. The drastic difference in defect migration activity in np Au irradiated at low and high dose rate. Compared to low dose rate, both defect generation rate and recombination rate increased prominently at higher dose rate.
